# Population Structure of the Greenhouse Whitefly, *Trialeurodes vaporariorum* (Westwood), an Invasive Species from the Americas, 60 Years after Invading China

**DOI:** 10.3390/ijms150813514

**Published:** 2014-07-31

**Authors:** Rui-Rui Gao, Wen-Ping Zhang, Huai-Tong Wu, Rui-Ming Zhang, Hong-Xu Zhou, Hui-Peng Pan, You-Jun Zhang, Judith K. Brown, Dong Chu

**Affiliations:** 1 Key Lab of Integrated Crop Pest Management of Shandong Province, College of Agronomy and Plant Protection, Qingdao Agricultural University, Qingdao 266109, China; E-Mails: gaoruirui2014@126.com (R.-R.G.); wenpingzhang@126.com (W.-P.Z.); zhangruiming0825@126.com (R.-M.Z.); hxzhou@qau.edu.cn (H.-X.Z.); 2 Department of Plant Protection, Institute of Vegetables and Flowers, Chinese Academy of Agricultural Sciences, Beijing 100081, China; E-Mails: huaitongwu@gmail.com (H.-T.W.); hppan0623@sina.com (H.-P.P.); 3 School of Plant Sciences, University of Arizona, Tucson, AZ 85721, USA; E-Mail: jbrown@ag.arizona.edu

**Keywords:** biological invasion, exotic introduction, genetic differentiation, genetic structure

## Abstract

Though the greenhouse whitefly, *Trialeurodes vaporariorum* (Westwood) (Hemiptera: Aleyrodidae) was introduced into China more than 60 years ago, the genetic diversity and structure of this exotic insect pest and virus vector have not been studied. To investigate the population genetic characteristics of this invasive species and to identify potential invasion routes, the genetic diversity and population structure of 17 collections of *T. vaporariorum* from nine provinces in China were analyzed using seven microsatellite loci. The results of the analyses indicated that the genetic diversity for the populations examined from the four provinces: Jilin, Ningxia, Guizhou and Qinghai, was lower than the genetic diversity of populations from the five provinces: Yunnan, Shandong, Shanxi, Liaoning, and Gansu. The *T. vaporariorum* populations analyzed in this study grouped as two distinct genetic clusters based on the analysis using STRUCTURE, whereas, 8 clusters were identified based on the BAPS analysis. Of the 136 genetic distance (*Fst*) values, 128 (94%) were associated with a significant exact test. There was a significant relationship between *Fst* and geographical distance. These results demonstrate that populations of *T. vaporariorum* in China exhibit significant genetic differentiation, indicating the likelihood that multiple introductions of *T. vaporariorum* into China have occurred. Also, the populations collected from the provinces of Jilin, Ningxia, Guizhou and Qinghai appear to represent secondary introductions originating from other Chinese provinces.

## 1. Introduction

The greenhouse whitefly, *Trialeurodes vaporariorum* (Westwood), which has its origin in tropical or subtropical America (Mound & Halsey, 1978), is an economically important pest of horticultural and ornamental crops, worldwide [1]. It is distributed on all continents except Antarctica [2,3,4,5,6]. The greenhouse whitefly is a polyphagous species, colonizing more than 250 host plants [7,8]. It can be both a vector of plant viruses [9,10,11,12], and a pest, causing considerable damage to the plant by feeding in the phloem [1]. Under greenhouse conditions this pest can multiply quickly many generations [2], and in addition, this whitefly has a propensity to develop resistance to insecticides [13].

In China, *T. vaporariorum* was first discovered in Beijing during the 1940s. During the early 1960s, it was found in Tianjin, and then in Inner Mongolia in the 1970s [14]. Since the first serious outbreak of the greenhouse whitefly in fields and greenhouses in Beijing during 1976 [15], it has reached pest status, causing damage to food crop and ornamental plants in a number of provinces including Xinjiang and Gansu in the 1990s. Currently, the greenhouse whitefly is known to be distributed in at least 22 provinces of China [14]. Though the greenhouse whitefly has existed in China for more than 60 years, its genetic diversity and population structure have not been studied to understand the extent of genetic differentiation, the number of distinct invasive events, and the routes by which these invasions have occurred. Such information will aid in understanding the invasion biology and microevolution of the greenhouse whitefly in China.

Extensive studies using molecular markers have provided estimates of the potential invasion routes, and of genetic characteristics of a number of invasive species [16,17,18]. For example, analysis of the genetic structure of the western flower thrips, *Frankliniella occidentalis* (Pergande) has revealed the invasion route and potential source of a secondary introduction in China [19], which could be useful for avoiding further spread and/or additional introductions of this pest. Because introduced populations usually experience rapid genetic differentiation in new environments, genetic structure analysis can lend insights into the genetic characteristics, and the degree of expansion or contraction that has occurred in invasive species populations [20,21]. Microsatellite loci have been used to study the genetics of insect populations because they are widely distributed in the genome, are inherited codominantly, exhibit abundant variation, and yield reproducible results [19,22,23,24].

The objective of this study was to determine the genetic structure and subsequent spread of the greenhouse whitefly, *T. vaporariorum* within China. To accomplish this, the genetic structure of 17 populations collected from nine provinces in China was analyzed using seven microsatellite loci.

## 2. Results and Discussion

### 2.1. Genetic Diversity

The values of genetic diversity indexes of the Chinese populations are provided in Table 1. The average number of alleles per locus (*Na*) ranged from 2.1429 (Xining, Qinghai) to 5.000 (Yuxi, Yunnan), and the effective number of alleles (*Ne*) ranged from 1.4428 (Xining, Qinghai) to 2.4447 (Yuxi, Yunnan). The expected heterozygosity (*He*) ranged from 0.2324 (Changchun, Jilin) to 0.5061 (Yuxi, Yunnan), while the observed heterozygosity (*Ho*) ranged from 0.1068 (Changchun, Jilin) to 0.4550 (Xishuangbanna, Yunnan). The value of *He* in each population was higher than the value of *Ho*. Nei’s expected heterozygosity (*Nei*) ranged from 0.2276 (Changchun, Jilin) to 0.4945 (Yuxi, Yunnan). 

The population of Yuxi, Yunnan (YN3) had the highest value of *He*, at 0.5061, while the population of Changchun, Jilin (JL3) had the lowest value at 0.2324. The populations from Yunnan (YN1-YN3), Shandong (SD), Shanxi (SX), Liaoning (LN), and Gansu (GS1-GS2) exhibited a *He* value of greater than the mean value of *He* 0.3683. In contrast, the *He* values of the other 9 populations from four provinces (Jilin, Ningxia, Guizhou, and Qinghai) were lower.

### 2.2. Analyses of Genetic Structure within Populations

The estimator of the fixation index, *Fis*, was not significant, indicating that there was no sub-structure within all populations (Table 1). In testing for deviation from mutation-drift equilibrium in BOTTLENECK software [25], the significant heterozygosity deficiency was not detected in the 17 populations. Significant heterozygosity excess was not detected in any population under either the TPM (two-phase mutation model) or SMM (stepwise mutation model), respectively. However, using the IAM (infinite allele model) a significant heterozygosity excess (*p* < 0.05) was detected in the six populations: QH1, QH2, GS1, YN1, YN2, and JL1 (Table 2), indicating that they may have undergone a genetic bottleneck.

### 2.3. Analyses of Genetic Structure among Populations

**Figure 1:**
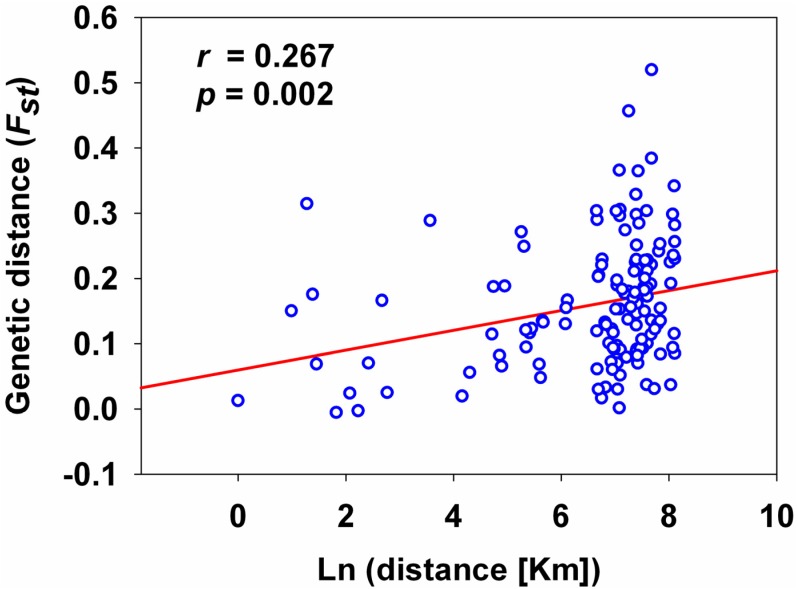
Relationship between genetic distance and log of geographical distance for *Trialeurodes vaporariorum*. The red line represents the regression line and blue circles represent the logarithm transformation of distance.

The analysis using MICRO-CHEKCER software [26] showed that only two of the seven loci had low frequencies of the null alleles. The estimated null alleles frequency ranged from 0.09 to 0.19 among all populations. When considering each population pair, 128 of 136 *Fst* values (94%) were associated with a significant test (Table 3). Estimates of pairwise *Fst* values ranged from −0.0057 (GS1/GS2) to 0.5194 (QH3/JL3). Obvious variation in allelic diversity and heterozygosity was found among the different populations (Table 1). Evidence of isolation by distance was found, based on the Mantel test for correlation between pairwise *Fst* and geographic distance (*r* = 0.267; *p* = 0.002) (Figure 1).

**Table 1:** Collection sites, population codes, dates of collection, host plants, and genetic diversity indexes for the greenhouse whitefly *Trialeurodes vaporariorum* populations from China examined in this study.

Locality	Code	Date	Host	*N*	*Na*	*Ne*	*Ho*	*He*	*Nei*	*Ar*	*Fis*	*p Values*	*Pwil*
Xining, Qinghai	QH1	2011.8	Eggplant	25	2.2857	1.6987	0.3200	0.3413	0.3345	2.0350	−0.0094	0.4250	0.9844
Xining, Qinghai	QH2	2012.9	Eggplant	25	2.1429	1.5074	0.3029	0.3144	0.3081	1.9549	0.0460	0.5966	0.9453
Xining, Qinghai	QH3	2012.9	Kidney bean	25	2.1429	1.4428	0.2229	0.2499	0.2449	1.7926	0.1648	0.3768	0.6875
Baiyin, Gansu	GS1	2011.8	Tomato	25	2.5714	1.8688	0.3143	0.3774	0.3698	2.2439	0.1919	0.1261	0.8906
Baiyin, Gansu	GS2	2011.8	Eggplant	25	2.8571	1.7495	0.3386	0.3849	0.3771	2.2721	0.0916	0.2804	0.5000
Yinchuan, Ningxia	NX	2012.9	Eggplant	24	3.1429	1.5985	0.2229	0.3277	0.3208	2.2344	0.3338	0.2016	0.0547
Xishuangbanna, Yunnan	YN1	2011.9	Tomato	27	3.0000	1.9985	0.4308	0.4563	0.4478	2.5229	0.0496	0.2631	0.9219
Xishuangbanna, Yunnan	YN2	2011.9	Eggplant	27	3.0000	2.1305	0.4550	0.4836	0.4746	2.6001	0.0329	0.4914	0.9453
Yuxi, Yunnan	YN3	2012.1	Kidney bean	22	5.0000	2.4447	0.2993	0.5061	0.4945	3.6000	0.4247	0.1700	0.1484
Guiyang, Guizhou	GZ1	2011.7	Tomato	27	2.5714	1.6685	0.4011	0.3651	0.3583	2.2656	−0.0462	0.4125	0.9219
Guiyang, Guizhou	GZ2	2011.8	Eggplant	24	3.1429	1.5665	0.3003	0.3121	0.3055	2.2867	−0.0203	0.5758	0.0156
Yuncheng, Shanxi	SX	2011.1	Tomato	12	3.4286	2.0678	0.3693	0.4408	0.4189	3.0106	0.1476	0.1863	0.3438
Jinan, Shandong	SD	2012.9	Cucumber	25	4.4286	1.9886	0.3026	0.4307	0.4219	3.0181	0.2721	0.1771	0.0195
Fushun, Liaoning	LN	2011.1	Tomato	27	3.4286	1.8539	0.2116	0.3700	0.3631	2.5496	0.2853	0.2208	0.2891
Changchun, Jilin	JL1	2012.8	Tomato	24	2.4286	1.6735	0.3323	0.3495	0.3421	2.1044	0.0699	0.3141	0.9453
Changchun, Jilin	JL2	2012.8	Cucumber	24	3.0000	1.7421	0.2307	0.3181	0.3113	2.3799	0.2063	0.1560	0.2813
Changchun, Jilin	JL3	2012.9	Pepper	25	2.4286	1.4949	0.1068	0.2324	0.2276	2.0003	0.5812	0.0505	0.0781
Mean	-	-	-	-	3.0000	1.7938	0.3036	0.3683	0.3600	2.4042	-	-	-

For each sample, the following genetic diversity indexes are indicated: sampling site, population code, date of collection, host plant, sample size (*N*), average number of alleles per locus (*Na*), the effective number of alleles (*Ne*), the observed heterozygosity (*Ho*), the expected heterozygosity (*He*), Nei’s expected heterozygosity (*Nei*), allelic richness (*Ar*), estimator of the fixation index (*Fis*), and the Wilcoxon test *p* value for heterozygosity deficit, compared to expectations at mutation-drift equilibrium (*Pwil*).

**Table 2:** Comparison of the within-population tests for heterozygosity excess tests using the models IAM (infinite allele model), TPM (two-phase mutation model), and SMM (stepwise mutation model) in BOTTLENECK.

Locality	Population Code	Heterozygosity Excess *p* Values		
		IAM	TPM	SMM
Xining, Qinghai	QH1	**0.0313**	0.0313	0.1094
Xining, Qinghai	QH2	**0.0234**	0.0781	0.2813
Xining, Qinghai	QH3	0.3125	0.4063	0.8906
Baiyin, Gansu	GS1	**0.0469**	0.3125	0.4063
Baiyin, Gansu	GS2	0.3438	0.5781	0.5781
Yinchuan, Ningxia	NX	0.7109	0.9609	0.9883
Xishuangbanna, Yunnan	YN1	**0.0391**	0.2188	0.2813
Xishuangbanna, Yunnan	YN2	**0.0195**	0.1484	0.3438
Yuxi, Yunnan	YN3	0.7656	0.9453	0.9883
Guiyang, Guizhou	GZ1	0.0547	0.2188	0.4219
Guiyang, Guizhou	GZ2	0.9453	0.9922	1.0000
Yuncheng, Shanxi	SX	0.4219	0.7188	0.9453
Jinan, Shandong	SD	0.9453	0.9883	0.9922
Fushun, Liaoning	LN	0.7109	0.7656	0.9727
Changchun, Jilin	JL1	**0.0234**	0.0781	0.5000
Changchun, Jilin	JL2	0.5781	0.7813	0.9609
Changchun, Jilin	JL3	0.7188	0.9453	0.9922

Bolded numbers indicate they are significant at *p* < 0.05.

**Table 3:** Population pairwise *Fst* (genetic distance) values.

Population Code	QH1	QH2	QH3	GS1	GS2	NX	YN1	YN2	YN3	GZ1	GZ2	SX	SD	LN	JL1	JL2
QH2	**0.1661**	-	-	-	-	-	-	-	-	-	-	-	-	-	-	-
QH3	**0.2883**	**0.3145**	-	-	-	-	-	-	-	-	-	-	-	-	-	-
GS1	**0.1164**	**0.1208**	**0.2489**	-	-	-	-	-	-	-	-	-	-	-	-	-
GS2	**0.1232**	**0.0943**	**0.2711**	−0.0057	-	-	-	-	-	-	-	-	-	-	-	-
NX	**0.1664**	**0.1549**	**0.1301**	**0.1352**	**0.1327**	-	-	-	-	-	-	-	-	-	-	-
YN1	**0.1615**	**0.2246**	**0.2508**	**0.0896**	0.0877	**0.2003**	-	-	-	-	-	-	-	-	-	-
YN2	**0.1282**	**0.2148**	**0.2978**	**0.0913**	**0.0928**	**0.2275**	0.0126	-	-	-	-	-	-	-	-	-
YN3	**0.1463**	**0.0699**	**0.2289**	**0.0826**	**0.0816**	**0.1499**	**0.1755**	**0.1503**	-	-	-	-	-	-	-	-
GZ1	**0.1529**	**0.0015**	**0.2958**	**0.1144**	**0.0969**	**0.1768**	**0.2045**	**0.2025**	**0.0609**	-	-	-	-	-	-	-
GZ2	**0.3056**	**0.0517**	**0.3656**	**0.1973**	**0.1872**	**0.2737**	**0.2900**	**0.3035**	**0.1195**	0.0248	-	-	-	-	-	-
SX	**0.0703**	**0.0856**	**0.1530**	**0.0723**	**0.0603**	**0.0907**	**0.1326**	0.1289	**0.0331**	**0.0817**	**0.1879**	-	-	-	-	-
SD	**0.1891**	**0.0301**	**0.3029**	**0.1223**	**0.1169**	**0.1830**	**0.2293**	**0.2204**	**0.0165**	**0.0194**	**0.0558**	**0.1008**	-	-	-	-
LN	**0.1002**	**0.0370**	**0.3036**	**0.0929**	**0.1064**	**0.1698**	**0.2250**	**0.1922**	**0.0368**	**0.0309**	**0.1221**	**0.0790**	**0.0238**	-	-	-
JL1	**0.1914**	**0.1139**	**0.3838**	**0.1726**	**0.1855**	**0.2842**	**0.2562**	**0.2301**	**0.0848**	**0.0837**	0.1541	**0.1784**	0.0300	**0.0478**	-	-
JL2	**0.1803**	**0.1370**	**0.4562**	**0.1864**	**0.1819**	**0.3284**	**0.2981**	**0.2354**	**0.0939**	**0.1298**	**0.2418**	**0.1562**	**0.0916**	**0.0654**	**0.0686**	-
JL3	**0.2210**	**0.1356**	**0.5194**	**0.2280**	**0.2118**	**0.3644**	**0.3415**	**0.2817**	**0.1150**	**0.1351**	**0.2527**	**0.2109**	**0.0936**	**0.0684**	**0.0703**	−0.0031

Significant values (*p* < 0.05) for pairwise *Fst* are in bold.

**Figure 2:**
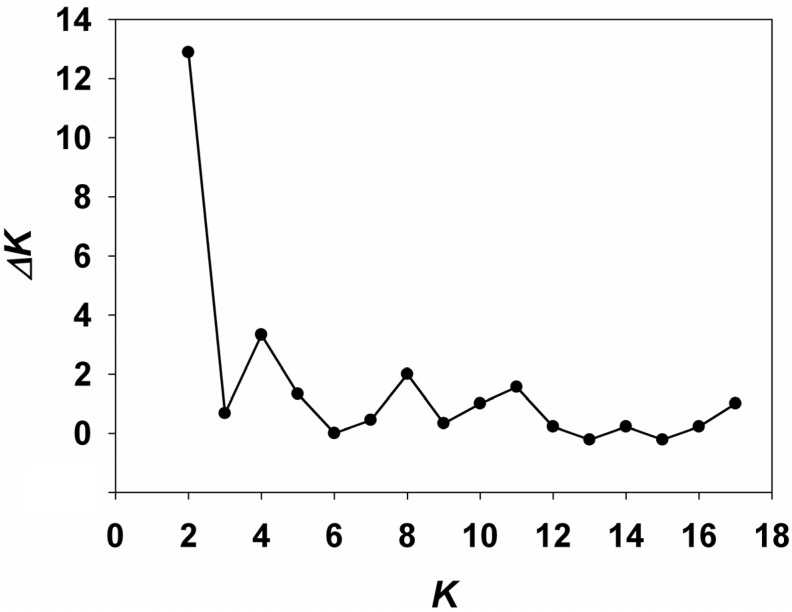
Scatter plots of Δ*K*.

**Figure 3:**
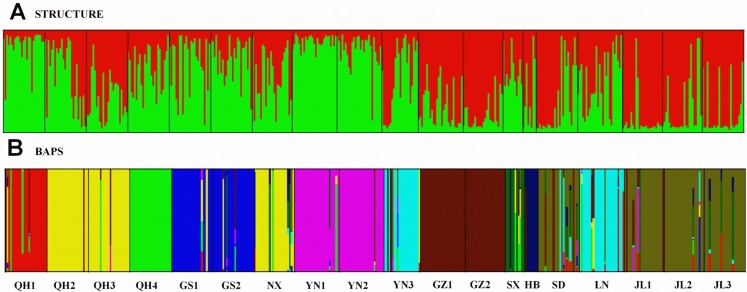
Results of genetic clustering using STRUCTURE (**a**) and BAPS (**b**) analysis, based on the 7 microsatellite loci used in the study.

The optimal number of clusters obtained with Evanno’s Δ*K* method was two (Figure 2 and Figure 3a). Thus, analyses using STRUCTURE software [27] identified two distinct genetic clusters. Eight populations (NX, GS1, GS2, SX, QH1, QH3, YN1, YN2) formed one cluster, and nine populations (JL1, JL2, JL3, LN, SD, QH2, YN3, GZ1, GZ2) formed another cluster. Analyses using BAPS software [28,29] identified ten genetic clusters overall (Figure 3b), with three clusters consisting of only one population each: QH3, SX, and SD, respectively; the three populations QH1, QH2, and NX formed one cluster, the populations GS1 and GS2 formed one cluster, YN1 and YN2 formed one cluster, YN3 and LN formed one cluster, and GZ1 and GZ2 formed one cluster.

### 2.4. Discussion

An investigation of the genetic diversity and structure of *T. vaporariorum* populations introduced into China beginning at least 60 years ago, has revealed new information about the genetic diversity and structure, based on the analysis of 17 populations from nine provinces using seven microsatellite loci. The results indicated that the genetic diversity for the populations from Jilin, Ningxia, Guizhou, and Qinghai was lower than for those from Yunnan, Shandong, Shanxi, Liaoning, and Gansu. *T. vaporariorum* populations formed two genetic clusters based on STRUCTURE analysis, and eight clusters based on BAPS analysis. The discrepancy in clustering between STRUCTURE and BAPS may have been due to the underlying model of admixture implemented by each program. Both genetic clustering analyses showed that Chinese populations of *T. vaporariorum* have significant genetic differentiation, which indicates multiple introductions of *T. vaporariorum* have occurred into China. The invasion pattern involving multiple introductions has been revealed in many invasive species including mosquitofish *Gambusia* spp., ctenophore *Mnemiopsis leidyi*, and caprellid *Caprella scaura* [30,31,32]. Multiple introductions are regarded as one main source for genetic diversity [33], which is often associated with successful invasions of some species [34,35]. Thus, we speculate that multiple introductions of *T. vaporariorum* have occurred into China, which might have been helpful to its successful invasion.

Of the 136 *Fst* values, 128 (94%) were associated with a significant exact test. However, the significant correlation was observed between *Fst* and geographical distance, suggesting that the spread of the populations was most likely associated with natural dispersal. The *Fst* data and analyses support the hypothesis that the introduced populations represent several distinct genetic lineages. Three possible explanations can be considered to explain the genetic differentiation of *T. vaporariorum* populations in China. One possible explanation could be a low rate of gene flow among the populations, which is known to result in the rapid genetic differentiation. The second possibility is that whitefly experienced rapid evolution to facilitate adaptation to new environments following the introduction. The genetic drift and natural selection resulting from exposure to some new plant hosts, climate, and management practices, all features that differ among regions in China, may have resulted in the selection of different variants [20].

The genetic diversity analysis based on *He* values indicates that the populations from four provinces: Jilin, Ningxia, Guizhou, and Qinghai might represent a secondary introduction stemming from the Yunnan, Shandong, Shanxi, Liaoning, and Gansu populations. When the results are considered together with the STRUCTURE and BAPS analysis, Jilin populations appear to have come from Shandong, whereas, the Ningxia population is more closely associated with Qinghai. However, it is not clear which population represents the primary introduction. Further, an association between a single population pair (the LN population from Liaoning and YN population from Yunnan) was observed, which can be explained by long distance transport of infested plant materials.

The significant deviation from Hardy-Weinberg equilibrium (HWE) could have arisen from inbreeding, subpopulation structure, and/or null alleles. Null alleles can be ruled out as an explanation in this study. Our study revealed that only two of the seven loci had low frequencies of the null alleles and the estimated null allele frequency ranged from 0.09 to 0.19 among all populations. Dakin and Avise [36] concluded that when the null alleles frequencies are less than 0.2, their presence causes a slight underestimate of the average exclusion probability at a locus. Thus, the null allele in our study is unlikely to appreciably influence Hardy-Weinberg equilibrium. The test for deviation from mutation-drift equilibrium suggested that bottleneck effects did not contribute importantly during the genetic differentiation of *T. vaporariorum* in China. This is based on the observation that only 6 of the 17 populations exhibited significant heterozygosity excess, the signature of a bottleneck (under the IAM model), further because heterozygosity excess was not predicted under either the TPM or SMM models.

Overall, the genetic diversity and the distinct genetic structure revealed by the cluster analysis suggest the likelihood of multiple introductions into China since the initial introduction of this exotic species 60 years ago.

## 3. Experimental Section

### 3.1. Field Sampling

**Figure 4:**
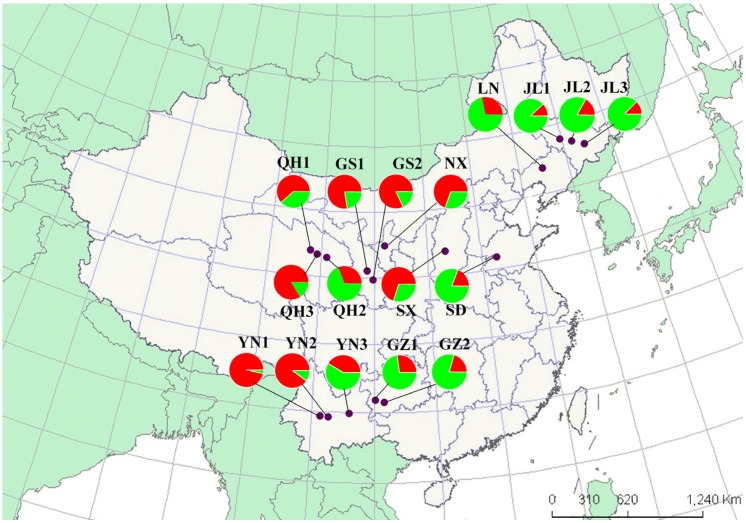
The geographical distribution of the populations and the genetic structure of the *Trialeurodes vaporariorum* revealed by STRUCTURE analysis (Figure 3a). The codes are listed in Table 1. QH1, QH2, and QH3 represent three populations from Qinghai. GS1 and GS2 represent two populations from Gansu. YN1, YN2, and YN3 represent three populations from Yunnan. GZ1 and GZ2 represent two populations from Guizhou. JL1, JL2 and JL3 represent three populations from Jilin. NX, SX, SD, and LN represent the population from Ningxia, Shanxi, Shandong, and Liaoning, respectively.

Adult *T. vaporariorum* were collected from cucumber, eggplant, and kidney bean plants in nine provinces throughout China during 2011–2012 (Figure 4). The sampling locations are shown in Figure 4. At least 100 adult whitefly specimens were collected from whitefly-colonized plants at each location. A total of 413 individuals from 17 populations (average sample size of 24 individuals ranging from 12 to 27 per population) were genotyped. The host plants and sample sizes per location are listed in Table 1. The whitefly specimens were stored in 95% ethanol at −20 °C until the DNA was extracted.

### 3.2. DNA Extraction and Microsatellite Genotyping

Genomic DNA was extracted from each individual adult *T. vaporariorum* using the DNAzol kit (Molecular Research Center, Inc., Cincinnati, OH, USA), and stored at −20 °C. The *T. vaporariorum*-specific primers (the forward primer CO1-F: 5'-GCCTGGTTTTGGCATTA-3', and the reverse primer CO1-R: 5'-GCTTATTTAGCACCCACTCTA-3') were used to amplify the mitochondrial cytochrome oxidase I gene fragment (752 bp in size) to determine the identity of each whitefly individual (patent number is CN201310192726.5), resulting in the confirmation of 413 individuals as the whitefly *T. vaporariorum*. The PCR reactions were performed in 20 μL buffer containing 2 μL 10× buffer, 1.5 mM MgCl_2_, 0.2 μM dNTPs, 1 unit Taq DNA polymerase, 2 μL template DNA and 0.2 μM of each primer. PCR amplification was carried out as follows: initial denaturation at 94 °C for 5 min, followed by 35 cycles of 30 s at 94 °C, 30 s at 50 °C, and 60 s at 72 °C, and a final elongation step at 72 °C for 30 min.

Seven pairs of microsatellite primers (code: Tvap-1-2, Tvap-2-2C, Tvap-3-3, Tvap-3-2, Tvap-1-1C, Tvap-1-3, and Tvap-1-4) were used to amplify the microsatellite loci using *T. vaporariorum* DNA as the template [37]. The primers and the annealing temperature were described in Table 4. The PCR reactions were performed in 20 μL buffer containing 2 μL 10× buffer, 1.5 mM MgCl_2_, 0.2 μM dNTPs, 1 unit Taq DNA polymerase, 2 μL template DNA and 0.2 μM of each primer. PCR amplification was carried out as follows: initial denaturation at 94 °C for 4 min, followed by 35 cycles of 30 s at 94 °C, 90 s at the primer-specific annealing temperature (Table 4) and 60 s at 72 °C, and a final elongation step at 72 °C for 30 min. The PCR products were run on an ABI 3730xl DNA analyzer (Sangon, Shanghai, China) and the allele size was determined by comparing the mobility of the PCR products to the GeneScan™ 400HD size standard using GeneMapper software version 3.2 (Applied Biosystems, Shanghai, China). 

**Table 4:** The seven pairs of microsatellite primers used in this study (as previously described in Ovcarenko *et al.* [37]).

Code	GenBank Number	Primer (5'–3')	Annealing Temperature (°C)	Range (bp)
Tvap-1-2	GF112025	PrimerA: CTGTGAATCCCTCAGAAATC PrimerB: TGACCTCTCTCAGGCTTTTA	57	180–238
Tvap-2-2C	GF112021	PrimerA: CTGAAAGTCTTATTAGAGCC PrimerB: CTAACTGATTCCATAGTCG	55	150–220
Tvap-3-3	GF112019	PrimerA: CGCAAATCATACTTCCTTTC PrimerB: AAATACAGGCGACTCATGTC	55	222–238
Tvap-3-2	GF112017	PrimerA: GGAGGTCATTACTCATTTCG PrimerB: CATAAATTTTCGGCTCACTC	55	176–184
Tvap-1-1C	GF112015	PrimerA: GAGACTCCACGATGTCTGTC PrimerB: TTCCCCTATCGTATGTTCAC	57	193–233
Tvap-1-3	GF112026	PrimerA: TATAGGGGTGTTGTGGTGTT PrimerB: CGCTACCAAATCGTAATTAC	55	147–197
Tvap-1-4	GF112020	PrimerA: GATTTAGCCCAGTTCATTTG PrimerB: CTTCAGTTGAGCTGCTGATG	55	218–268

### 3.3. Analyses of Genetic Diversity

For each of the 17 populations of *T. vaporariorum*, the average number of alleles per locus (*Na*), the effective number of alleles (*Ne*), the observed heterozygosity (*Ho*), the expected heterozygosity (*He*), and Nei’s expected heterozygosity (*Nei*) were calculated using POPGENE v.1.31 [38]. The program FSTAT 2.9.3.2 was used to calculate allelic richness (*Ar*) [39].

### 3.4. Analyses of Genetic Structure within Populations

The Weir and Cockerham’s *Fis* [40] within each population was quantified with GENEPOP v.3.4 software [41]. Conformity to Hardy-Weinberg equilibrium was assessed with exact tests in GENEPOP v.3.4 with Markov chain parameters of 10,000 de-memorization steps, 1000 batches and 10,000 iterations per batch. To correct for multiple comparisons, a sequential Bonferroni correction was applied to both HWE tests. When deviation from HWE was detected, the presence of null alleles or/and scoring errors was estimated using MICRO-CHECKER [26].

Deviation of the mutation-drift equilibrium in the populations was tested using the approach in BOTTLENECK software [25]. The heterozygosity deficit is used to test for population expansion, whereas the heterozygosity excess test is used to provide evidence of a genetic bottleneck. Using the Wilcoxon test, the heterozygosity deficit was evaluated under the two-phase mutation model (TPM) recommended for microsatellite data [42]. The possibility of bottleneck events within the 17 populations was examined under three mutation models (Two Phase Mutation Model (TPM), Infinite Allele Model (IAM), and Stepwise Mutation Model (SMM)) [25,42]. The TPM model was used with the default settings of 30% variation from the IAM model, and 70% from the SMM model.

### 3.5. Analyses of Genetic Structure among Populations

The traditional population differentiation approach was based on *Fst* values. The Weir and Cockerham’s estimator of the fixation index *Fst* [40] was calculated using GENEPOP v.3.4 [41]. The correlation between genetic differentiation and geographic distance was examined by Mantel test using IBDWS v.3.15 [43].The distribution of genetic variation was investigated by performing an analysis of molecular variance (AMOVA) using ARLEQUIN v.3.5 [44], and by calculating allelic diversity, heterozygosity, and pairwise values of *Fst* among 17 populations. The genetic clustering of samples was examined using STRUCTURE v.2.3.2 and BAPS v.4.14 [27,28,29]. Population structure analyzed using STRUCTURE v.2.3.2 employed the Bayesian clustering approach with a burn-in period of 50,000 iterations and one million Markov chain Monte Carlo (MCMC) repetitions under the admixture ancestry model. 20 independent runs were performed for each testing *K* value, ranging from *K* = 1 to 17, and Δ*K* was used to calculate the optimal number of genetic clusters (*K*) [45]. When estimating individual ancestry coefficients using BAPS v.4.14 [28,29], we used the recommended values that 100 iterations to estimate the admixture coefficients individual, 20 iterations to estimate the admixture coefficients for the reference individuals and 200 reference individuals from each population.

## 4. Conclusions

These results demonstrate that populations of *T. vaporariorum* in China exhibit significant genetic differentiation, indicating the likelihood that multiple introductions of *T. vaporariorum* into China have occurred. Also, the populations collected from the provinces of Jilin, Ningxia, Guizhou and Qinghai appear to represent secondary introductions originating from other Chinese provinces.
